# Current Methods of Magnetic Resonance for Noninvasive Assessment of Molecular Aspects of Pathoetiology in Multiple Sclerosis

**DOI:** 10.3390/ijms21176117

**Published:** 2020-08-25

**Authors:** Petra Hnilicová, Oliver Štrbák, Martin Kolisek, Egon Kurča, Kamil Zeleňák, Štefan Sivák, Ema Kantorová

**Affiliations:** 1 Biomedical Center Martin, Jessenius Faculty of Medicine in Martin, Comenius University in Bratislava, 036 01 Martin, Slovakia; oliver.strbak@uniba.sk (O.Š.); martin.kolisek@uniba.sk (M.K.); 2 Clinic of Neurology, Jessenius Faculty of Medicine in Martin, Comenius University in Bratislava, 036 01 Martin, Slovakia; egon.kurca@uniba.sk (E.K.); stefan.sivak@uniba.sk (Š.S.); ema.kantorova@uniba.sk (E.K.); 3 Clinic of Radiology, Jessenius Faculty of Medicine in Martin, Comenius University in Bratislava, 036 01 Martin, Slovakia; kamil.zelenak@uniba.sk

**Keywords:** multiple sclerosis, ^1^H MRS, ^31^P MRS, Mg^2+^, Fe deposits

## Abstract

Multiple sclerosis (MS) is an autoimmune disease with expanding axonal and neuronal degeneration in the central nervous system leading to motoric dysfunctions, psychical disability, and cognitive impairment during MS progression. The exact cascade of pathological processes (inflammation, demyelination, excitotoxicity, diffuse neuro-axonal degeneration, oxidative and metabolic stress, etc.) causing MS onset is still not fully understood, although several accompanying biomarkers are particularly suitable for the detection of early subclinical changes. Magnetic resonance (MR) methods are generally considered to be the most sensitive diagnostic tools. Their advantages include their noninvasive nature and their ability to image tissue in vivo. In particular, MR spectroscopy (proton ^1^H and phosphorus ^31^P MRS) is a powerful analytical tool for the detection and analysis of biomedically relevant metabolites, amino acids, and bioelements, and thus for providing information about neuro-axonal degradation, demyelination, reactive gliosis, mitochondrial and neurotransmitter failure, cellular energetic and membrane alternation, and the imbalance of magnesium homeostasis in specific tissues. Furthermore, the MR relaxometry-based detection of accumulated biogenic iron in the brain tissue is useful in disease evaluation. The early description and understanding of the developing pathological process might be critical for establishing clinically effective MS-modifying therapies.

## 1. Introduction

Multiple sclerosis (MS) is a multifactorial neurodegenerative disease that affects over 2.5 million individuals worldwide [1,2]. It is the most common cause of neurologic disability in young adults [3,4]. The traditional “outside-in” hypothesis of MS etiology is based on an autoimmune provenance whereby dysregulated auto-reactive T cells in the periphery cross into the central nervous system (CNS) and, together with macrophages and B cells, proceed to attack predominantly myelin elements [1,5,6]. Ongoing inflammation then results in a relapsing–remitting clinical course and fundamentally contributes to comprehensive CNS injury [4,7]. However, in recent years, an alternative “inside-out” MS-caused hypothesis that proposes initial multifunctional failures within the CNS has come to the fore. MS is suspected of being a primary degenerative disease linked with local inflammation, leading to the release of antigenic cell components such as myelin oligodendrocyte glycoprotein, myelin basic protein, and proteolipid protein [6,8,9]. However, its diagnosis and treatment strategies are complicated by the presence of disease heterogeneity with distinct courses and diverse therapy responses. Patients are diagnosed as having early MS (formerly clinicaly isolated syndrome suspected of being MS (CIS)), clinicaly definite multiple sclerosis (CDMS), relapsing-remitting multiple sclerosis (RRMS), progressive-relapsing MS (PRMS), and primary- and secondary-progressive multiple sclerosis (PPMS, SPMS) [2,4,10]. Moreover, the disease causes variable degrees of motor, sensory, behavioral, mental, emotional, and cognitive impairment [11,12,13].

MS is predominately characterized as an inflammatory disease of the CNS involving the initiation of myelin loss and of axonal and neuronal damage leading to progressive neurological dysfunction [8,14]. However, recent research has shown that the neurodegenerative process is not clearly related to inflammatory lesions but is instead associated with diffuse neuro-axonal loss caused by various pathological phenomena [9,14]. Some authors have suggested that a cell-mediated autoimmune response occurs against extraneous or even the body’s own components, with the consequent damage of oligodendrocytes and progressive axonal degeneration [10,15]. One mechanism of this neurodegeneration might be associated with the production of reactive oxygen species from activated microglia and macrophages with a subsequent inadequate energy supply to the CNS [9,16]. Morphological and biochemical changes have been described in the tissue in conjunction with an intracellular accumulation of iron ions suggesting the occurrence of programmed cell death-apoptosis [17,18]. Moreover, ferroptosis as non-apoptotic and iron-dependent programmed cell death is now being considered, since inflammatory demyelination is consistent with ferroptotic damage [19]. The excessive iron accumulation might be caused by slowing intracellular metabolism and by the aggravation of waste product removal [2,12]. However, whether the iron accumulation is the initial cause or a result of the pathology remains unknown. Neuronal dysfunction might also be initiated by enhanced glutamate neuro-excitation, so-called glutamate excitotoxicity [13,20]. In these specific circumstances, glutamate, an essential mediator of excitation, might be a co-founder of oxidative and metabolic stress and then contribute to neuronal damage in MS [12,21]. Furthermore, gray matter (GM) changes have been found to have a higher impact on the progression, physical disability, and cognitive impairment of MS in comparison with white matter (WM) demyelination [9,14,22]. Nevertheless, initial injuries to GM have been proposed to extend strongly in the early stage of MS [12,14]. In recent years, disease progression markers have been researched, particularly those suitable for the detection of early subclinical changes [18,23,24].

To date, magnetic resonance (MR) methods are considered to be the most sensitive and crucial in the diagnosis of MS [2,14,25]. In particular, MR spectroscopy (MRS) allows the noninvasive quantification of biomedically relevant metabolites, amino acids, and bioelements and their metabolic alternations in tissue in vivo [25,26]. Increasing importance has also been attached to MR imaging (MRI) techniques that have been shown to be highly sensitive in evaluating disease burden. Notably, iron accumulation can be detected in brain tissue based on altered T_2_ relaxation times [18,27]. In view of the expansion of new disease-modifying therapies available for MS treatment, an understanding of the pathological processes associated with disease progression has become important, as has the identification of new meaningful noninvasive radiological biomarkers for evaluating the clinical efficacy of treatments.

## 2. General MRI Techniques

For the clinical prognosis of MS, MR imaging techniques have an undisputed contribution, especially as a diagnostic tool for the visualization of MS lesions [28,29,30]. The gold standard for the detection of lesion load is T_2_-weighted MRI and fluid-attenuated inversion recovery (FLAIR) sequences. Currently, the preferable FLAIR provides a suppression of the signal from the cerebrospinal fluid and thus enables the better delineation of the periventricular and cortical/juxtacortical lesions at the expense of decreased lesion conspicuity in the posterior fossa or spinal cord compared with T_2_-weighted spin-echo sequences [28,29,30]. Furthermore, an excellent method suitable for the monitoring of disease activity manifesting as the appearance, recurrence, or extension of MS lesions is T_1_-weighed MRI after the intravenous injection of contrast medium, i.e., gadolinium (Gd)-enhanced MRI. Whereas the Gd-based contrast agents have revealed the breakdown of the blood–brain barrier in acute inflammatory lesions such as T_1_-hypointense (precontrast images)/T_1_-hyperintense (Gd-enhanced lesion) areas, the T_2_-hyperintense areas might indicate edema, gliosis, or axonal demyelination [28,31]. The evaluation of the contrast enhancement of T_1_-hypointensities enables pathological severity to be assessed [28,30]. Focal acute inflammatory regions are visible, which resolve over six months, in addition to persistent chronic lesions representing irreversible demyelination and axonal loss [28,30]. In the case of MS, such an assessment might be helpful for evaluating brain and spinal cord atrophy, which is usually quantified on T_1_-weighted images [29]. However, the annual rate of MS brain tissue loss is around 0.5–1% [28,29] and can vary during various destructive pathological processes, including demyelination, edema, neuro-axonal destruction, inflammation, or a reduction in water content. Generally, GM atrophy is more associated with MS progression and physical and cognitive disability than WM loss [28,29]. Furthermore, several adapted MRI techniques exist that allow a weighted MR-signal to be obtained that is based on disturbances in the structure (magnetization transfer MRI) and movements (diffusion MRI) of molecules or changes in the local tissue environment (susceptibility-weighted MRI), blood flow (perfusion MRI), and oxygenation (functional MRI), which can improve MS assessment (Table 1).

- Magnetization transfer MRI is based on the interactions that occur between free-water protons and protons bound to macromolecules and that are typically evaluated as their quantitative ratio/magnetization transfer ratio [29,32]. This technique reveals subtle brain tissue integrity loss during MS progression better than conventional MRI [28]. The magnetization transfer ratio has been demonstrated continuously to decrease in MS lesions and normal-appearing WM (NAWM) and has been related to the percentage of residual axons and the degree of myelin content [28,31].
- Diffusion-weighted MRI is a suitable method for the measurement of the water molecule motion in tissue, especially in the WM, in which water diffusion is preferably oriented along the axons and thus follows the WM tracts [29]. Any neuronal tract failure or axonal membrane permeability disruption should lead to an increase in the mean diffusivity (the averaged molecular motion) and to a decrease in the fractional anisotropy (the directional preponderance), as reported in chronic MS lesions and NAWM [28,31,33].
- Susceptibility-weighted MRI enhances the inhomogeneity of the magnetic field caused by the paramagnetic properties of venous deoxygenated hemoglobin and other nonheme iron, all of which affect the local magnetic susceptibility in the MS brain [29]. The highest potential of this technique is in the long-term monitoring of the disturbed microenvironment of MS lesions and veins, showing an increasing trend in the accumulation of iron deposits in these areas [28].
- Cerebral perfusion MRI provides information on capillary microcirculation in the tissue based on generating a blood flow contrast between multiple images by using a wide variety of blood-labeling methods such as exogenous tracers (Gd-contrast agent) or water protons from the arterial blood (arterial spin labeling) [32]. Perfusion studies reveal increased perfusion in acute inflammatory MS lesions, and thus vasodilation and decreased cerebral blood flow in non-enhancing persisting MS lesions [28,32]. Perfusion is also altered in NAWM and in cortical and subcortical GM, reflecting not only microvascular abnormalities, but also tissue degradation and blood–brain barrier permeability failure [28].
- Functional MRI enables the assessment of brain activation manifesting as differences in the deoxyhemoglobin concentration in the blood in activated brain areas, in response to the applied motor or visual stimuli [30,32]. Observed abnormalities in MS patients usually occur in the visual, cognitive, and motor systems with early manifestation, and tend to vary throughout the disease [28].

### Limitations of General MRI Techniques

Generally, MRI diagnostic techniques have a high sensitivity for MS lesion detection; however, they have a relatively poor pathological specificity and prognostic value [29,30]. Despite the T_2_-hyperintense lesion load and Gd-enhanced lesions being the cornerstones of MS diagnosis, they show only a weak correlation with clinical status, cognitive dysfunction, or neurological impairment [29,34]. Furthermore, the primary MRI diagnostic efficacy depends on precise image analysis and careful clinical presentation, utilizing the increasingly stringent MRI criteria for MS staging. Moreover, health risks have to be taken into account when using Gd-based contrast agents (i.e., nephrogenic systemic fibrosis, retention of Gd). Even though these materials are beneficial in MS diagnosis, a trend is apparent to introduce novel contrast agents (e.g., ultrasmall superparamagnetic particles of iron oxide, myeloperoxidase, gadofluorine M [29,32]). However, appropriate validation and safety guarantees have to be ascertained before their clinical application. Many methods other than conventional MRI techniques are being investigated in order to obtain a better correlation with the clinical course of the disease and a more detailed quantification of MS pathological mechanisms.

## 3. Magnetic Resonance Spectroscopy

MRS is a noninvasive alternative MR technique that enables characteristic tissue biomarkers to be explored in vivo without the need for a biopsy [25,26]. The MR phenomenon is based on the magnetic nature of atoms and their behavior in magnetic fields. The nuclei of atoms with an odd mass number possess the quantum property of “spin”, which can be understood as the nucleus spinning around its own axis, always aligning along the axis of a constant magnetic field [26,35]. The application of an electromagnetic impulse at a suitable “resonance” frequency causes nuclear excitation, i.e., stimulates transitions between the high and low energy states of nuclei. During the subsequent relaxation, radiofrequency signals are generated that can be expressed as a frequency spectrum [11,35]. Signals from individual metabolites, whose molecules contain MR-visible nuclei, appear as spectral peaks resonating at discrete frequencies shown in parts per million (ppm). The clinical applicability of MRS is given mainly by its detection sensitivity for the measured nucleus and by its biological occurrence in the tissue [11,26]. Therefore, proton (^1^H) and phosphorus (^31^P) MRS are the most useful MRS methods for brain examinations. When an MRS measurement is being planned, one of the fundamental decisions is whether to acquire data from a single voxel (so-called single-voxel spectroscopy (SVS) or localized spectroscopy) or multiple voxels (so-called chemical shift imaging (CSI) or magnetic resonance spectroscopic imaging (MRSI)). Whereas the SVS method relatively easily acquires data from a small, well-shimmed, and compact region in a relatively short scan time, providing spectra with a high signal-to-noise ratio, CSI collects spectroscopic data from multiple, much smaller, individual voxels, covering a larger, heterogeneous tissue mass in a single measurement [26,35,36,37].

### 3.1. ^1^H MRS

**Figure 1:**
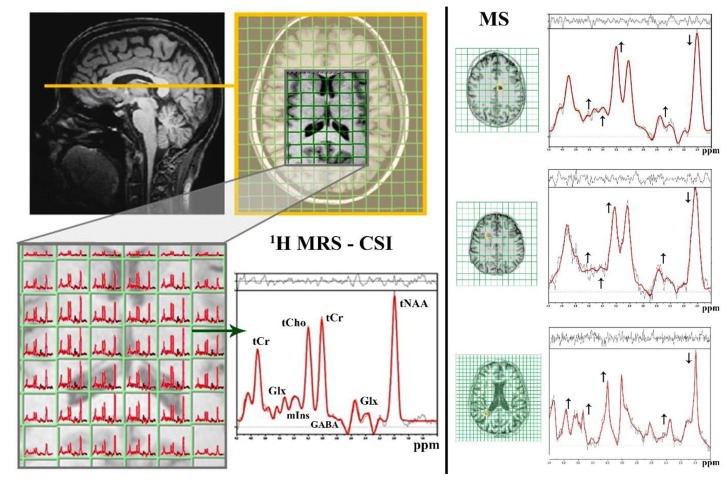
Visualization of proton-1 magnetic resonance spectroscopy (^1^H MRS). ^1^H MRS examination of brain tissue by using the multivoxel spectroscopy (^1^H magnetic resonance spectroscopic imaging (MRSI)–chemical shift imaging (CSI)) approach with the representing spectra (one CSI voxel indicated with the green arrow) containing metabolite peaks for total creatine (tCr), total choline (tCho), total N-acetyl-aspartate (tNAA), glutamate and glutamine (Glx), γ-aminobutyric acid (GABA), and myoInositol (mIns). The ^1^H MRS spectra for the healthy (on the left) and MS-affected (on the right) brain are also depicted together with the highlight of the typical metabolic peaks changes via small black arrows (↓: decreased, ↑: increased).

Various signals can be detected based on ^1^H MRS (Figure 1): the signal attributable to N-acetyl-aspartate (NAA) and N-acetyl-aspartyl-glutamate (NAAG), usually evaluated as the joint tNAA peak; the signal for creatine-containing compounds (i.e., creatine and phosphocreatine) referred as tCr; the signal from molecules contributing to the total choline (tCho) peak (i.e., phosphatidylcholine, glycerophosphatidylcholine, acetylcholine, and choline); the signal from myoInositol (mIns); the signals from neurotransmitters such as glutamate and glutamine (usually evaluated as one-peak Glx); and the signal from γ-aminobutyric acid (GABA) [11,13,25]. In general, approximately 25 additional compounds can be assessed throughout the brain: aspartate, glutathione, taurine, ethanolamine, histidine, glycogen, lactate (detectable only during pathological increment), or mobile lipids (i.e., triacylglycerol and cholesterol esters accumulated in lipid droplets) [26,37,38,39,40]. These are difficult to detect routinely; furthermore, only rarely are they suggestive of MS manifestations [3,41].

#### 3.1.1. Neuro-Axonal Degradation

**Figure 2:**
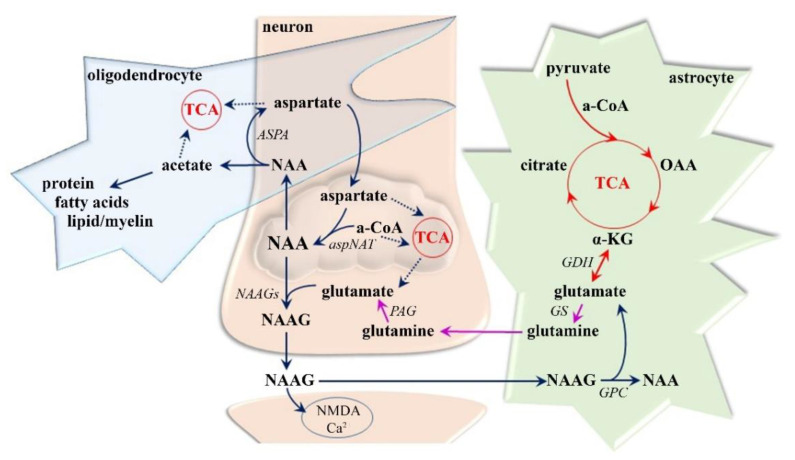
Schematic representation of N-acetyl-aspartate (NAA) metabolism. Acetyl coenzyme A (a-CoA) derived from pyruvate in neuronal mitochondria can be oxidized for adenosine triphosphate (ATP) production in the tricarboxylic acid (TCA) cycle or can be converted to citrate via citrate synthase or to NAA via aspartate N-acetyltransferase (aspNAT) in neuronal mitochondria. On the one hand, NAA is predominantly transported to oligodendrocytes for degradation via aspartoacylase (ASPA) and further use in protein, fatty acid/myelin lipid synthesis, and energy production. On the other hand, neuronal NAA can also combine with glutamate to produce N-acetylaspartylglutamate (NAAG) via NAAG synthetase (NAAGs). The latter is co-released from synaptic vesicles together with several neurotransmitters and can act on the glutamate receptor (N-methyl-D-aspartate (NMDA); presynaptic type 3 metabotropic glutamate receptors) where, via blocking, Ca^2+^ channels inhibit glutamate function. NAAG can also be hydrolyzed at the surface of astrocytes to NAA and glutamate via carboxypeptidase (GPC). In astrocytes, glutamate can, via glutamate dehydrogenase (GDH), be converted to α-ketoglutarate (α-KG) as part of the TCA cycle. Glutamate can also be combined with ammonia to produce glutamine via glutamine synthase (GS). Neurons can take up glutamine and use it as a source of glutamate via phosphate-activated glutaminase (PAG). Thus, NAA and glutamate can produce their own general circulation.

Despite NAA being one of the most concentrated (10–20 mM [42,43]) molecules in the CNS and despite its having been studied for more than five decades, its neurochemical functions, especially under pathological conditions, remain unclear [42,44]. As previously determined (Figure 2), NAA synthesis (via aspartate N-acetyltransferase) occurs exclusively in neuronal mitochondria, whereas NAA breakdown (via aspartoacylase) is a membrane-associated process localized predominantly in the oligodendrocytes [42,43,45]. Among its many biochemical features, NAA, is particularly important for the maintenance of neuro-glial signaling [42,44,46]. NAA has been proposed to be an intermediate metabolite in the formation of the not fully elucidated neurotransmitter NAAG [42,44,47], although NAAG reaches maximally 10% of the NAA concentration [43,46]. Therefore, NAA is thought to have a more crucial function as a reservoir for glutamate [43,47], which is the most abundant neurotransmitter in the human brain [12,13]. In particular, in times of dynamic glutamate replenishment, NAA can be converted to glutamate via dipeptide NAAG transformation (by carboxypeptidase expressed on the extracellular surface of astrocytes) [47,48] and an energetically favorable set of tricarboxylic acid (TCA cycle) reactions (α-ketoglutarate in neuronal mitochondria) [42,46]. Paradoxically, both NAA and NAAG are defined as (low affinity) agonists and antagonists at the main glutamate receptors, suggesting a primary role of NAA in avoiding myelin loss induced by glutamate overloading [48,49]. Moreover, NAA probably plays essential roles in neuronal osmoregulation, the secondary nitrogen removal system, mitochondrial bioenergetics maintenance, TCA cycle intermediate replacement, and lipid and protein synthesis including myelin formation in oligodendrocytes [41,43]. Given that NAA is considered to occur almost exclusively within neurons, axons, and dendrites [42,50], its peak in ^1^H MRS of the brain (Figure 1) is assumed to be a crucial biomarker of neuronal density and viability, a determinant of neuro-axonal functionality, and possibly an essential sign of irreversible disease progression [51,52].

The highest tNAA loss has been demonstrated to be the most marked in those patient groups with the most considerable neurological disability [25,50]. Furthermore, histopathology on biopsied brain tissues has confirmed reduced tNAA levels, notably in the acute phase of the disease [52,53]. Several MRS studies have reported a reduction in tNAA not only in acute MS lesions [50,51], but also in chronic MS lesions [25,54], indicating that axonal loss is characteristic in all types of MS lesions. However, decreased NAA levels have also been shown in other non-lesion brain areas such as NAWM and cortical GM in early RRMS [50,54], in the basal ganglia and thalamus in RRMS [55,56], and in the thalamus and hippocampus in PPMS [54,57]. Furthermore, a decline in tNAA ratios has been found in the hypothalamus of RRMS and SPMS and in early MS patients [24,52,58]. This is in agreement with observations of the cortical and subcortical demyelination that occurs during disease progression in GM structures, especially in the hippocampus, hypothalamus, thalamus, caudate, putamen, globus pallidus, and other structures of the basal ganglia [59,60,61]. Moreover, GM demyelination has recently been observed in biopsy material of an early MS patient, even before disseminated WM lesions become visible by MRI [5,7]. GM atrophy has been postulated to represent one of the earlier markers of degeneration in MS [62,63,64] and even seems to be a better marker of disease progression compared with whole-brain or the WM fraction [65,66,67], although factors other than demyelination might be responsible for GM atrophy. Axonal transection and neuronal, glial, and synaptic loss are also believed to be present in cortical GM lesions and might be responsible for cortical thinning in MS [9,60]. Furthermore, GM involvement has been demonstrated to precede WM damage and is associated with physical disability and cognitive impairment [22,64,65].

Based on ^1^H MRS, correlations have also been made between tNAA concentration and accompanying signs of the disease [50,68]. In particular, the reduction in tNAA in the frontal cingulate gyrus has been found to correlate with global memory functions in a group of early MS patients [60,69]. Lower tNAA in the tegmental pons of patients with RRMS has been associated with higher fatigue [70]. An inverse correlation of tNAA to disability has been observed in chronic lesions of RRMS, SPMS, and PPMS patients [25] and in the hypothalamus of patients in early MS stages [24]. Taken together, these data suggest that even a partial recovery of NAA levels can improve neuronal energetics, instigate remyelination, and support neuro-axonal integrity, thus relieving MS-associated symptoms. However, NAA-influencing medicaments (e.g., interferon beta-1b [71], glatiramer acetate [72], fluoxetine [73,74]) are still under examination.

#### 3.1.2. Demyelization and Reactive Gliosis

The altered mIns and tCho signals in ^1^H MRS throughout the brain (Figure 1) are associated with cell membrane pathophysiology, ongoing inflammation processes, and disrupted neuroglial connections, which are all essential signs of MS progression [24,50]. Variations in both peaks are often reported in progressive MS stages [25,51,75] but are not limited to MS lesions; they involve NAWM [76,77] and deep GM, particularly in the thalamus and hippocampus [52,57].

##### tCho—Cell Membrane Predictor

Whereas most of the tCho signals result from precursors (mainly phosphatidylcholine) and degradation products (mainly glycerophosphatidylcholine) of cell membrane phospholipids (especially the myelin sheet), tCho is generally recognized as a biomarker of cellular membrane integrity [24,78,79]. Studies focusing on MS have demonstrated increased tCho levels as a characteristic manifestation of demyelination or remyelination and as an accompanying sign during inflammation and gliosis in both WM and GM structures [67,79,80]. Elevated tCho values have been confirmed, especially in acute lesions, and also in the NAWM of progressive MS patients [78]. Furthermore, active demyelination, which is associated with profound acute axonal injury in all progressive MS forms (acute MS, RRMS, SPMS, PPMS), has been observed in deep GM structures (thalamus, hypothalamus, caudate nucleus, pallidum, and putamen) [9,63,81]. Membrane breakdown products themselves have, moreover, been postulated to complicate the regeneration of the impaired GM tissue and thus to worsen the MS manifestation [24,25]. However, the GM areas have revealed lower tCho overloading in RRMS and SPMS patients than in CIS and CDMS patients [51,52,54]. The initial active GM demyelination is, therefore, thought to be suppressed in progressive MS stages [24]. This idea explains the lack of differences in tCho throughout GM regions (i.e., thalamus, hippocampus, cortical GM) in RRMS patients or even the decreased tCho levels in progressive MS stages [56,58,82].

##### mIns—Glial Marker

**Figure 3:**
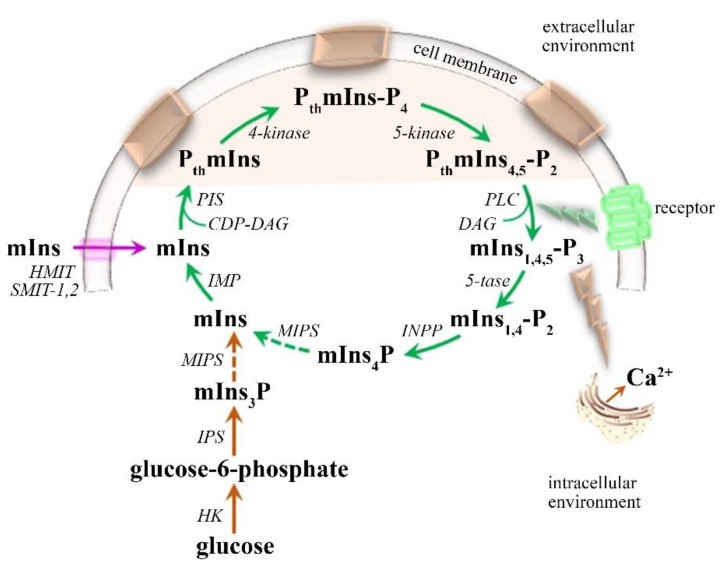
Schematic representation of myoInositol (mIns) metabolism. mIns can be incorporated into cytidine diphosphate diacylglycerol (CDP-DAG) via the activity of phosphatidylinositol synthase (PIS) generating phosphatidylmyoInositol (P_th_mIns). Further phosphorylation of P_th_mIns by phosphatidylinositol 4-kinase (4-kinase) forms phosphatidylmyoInositol 4-phosphate (P_th_mInsP_4_) and subsequently, via phosphatidylinositol 4-phosphate 5-kinase (5-kinase), produces phosphatidyl 4,5-bisphosphate (P_th_mIns_4,5_-P_2_). P_th_mIns, P_th_mInsP_4_ and P_th_mIns_4,5_-P_2_ are incorporated constituents of cell membranes (depicted as blocks in the cell membrane and colorfully highlighted in the intracellular environment). In response to agonists, activated receptors stimulate phospholipase C (PLC), which hydrolyzes P_th_mIns_4,5_-P_2_ to form DAG and myoInositol 1,4,5-trisphosphate (mIns_1,4,5_-P_3_). mIns_1,4,5_-P_3_ is a soluble molecule capable of binding to membrane receptors on the endoplasmic reticulum causing Ca^2+^ release into the cytoplasm. mIns_1,4,5_-P_3_ may also be dephosphorylated by inositolpolyphosphate 5-phosphatase (5-tase) forming myoInositol 1,4-bisphosphate (mIns_1,4_-P_2_) and further by inositol polyphosphate 1-phosphatase (INPP) generating myoInositol 4-monophosphate (mIns_4_P). The activity of several myoInositol phosphate synthases (MIPS) leads to the production of myoInositol 1-monophosphate (mInsP). The dephosphorylation of mInsP to mIns is catalyzed by inositol monophosphatase (IMP). However, mIns can be reuptaken from the extracellular space via three specialized mIns transporters (Na^+^-dependent myoInositol cotransporters-1 and 2 (SMIT-1,2); H^+^-dependent myoInositol cotransporter (HMIT)) or synthesized from glucose via hexokinase (HK) and myoInositol-3-phosphate-synthase (IPS) to myoInositol-3-phosphate (mIns_3_P) and further catalyzed by MIPS and IMP.

The level of mIns in the brain is primarily maintained (Figure 3) by the recycling of inositol-containing phospholipids that are tightly linked to the phosphatidylinositol pathway cycle (PIP cycle) [83,84,85], although, a small amount of brain mIns can be synthesized not only from glycolysis through glucose-6-phosphate (by inositol synthase and phosphatase) [83,84,85], but also by reuptake from the extracellular fluid (3% of mIns levels) via three specialized mIns transporters (Na^+^-dependent myoInositol cotransporters-1 and 2; H^+^-dependent myoInositol cotransporter) [84,85,86]. Among its important roles in CNS, the most prominent mIns functions are reported to be the synthesis of inositol-containing membrane phospholipids, protein phosphorylation, cellular osmoregulation, and glucose homeostasis maintenance [83,85,86]. Furthermore, mIns has been established as an important growth-promoting factor and co-factor of enzymes and as a messenger molecule in signal transduction [83]. Not only mIns, but also other components of the PIP cycle might be involved in a potential signaling mechanism influencing several cellular functions [85,86]. In particular, mIns is responsible for Ca^2+^ release from the endoplasmic reticulum and mitochondria, thereby raising the cytosolic Ca^2+^ concentration [83,86]. Furthermore, the bilateral functional relationship between inositol-1,4,5-triphosphate and the main neuronal glutamate receptors [85,86] have been declared to be responsible for enhanced excitatory neurotransmission and thus the depolarization-induced intracellular Ca^2+^ increase. This synergic depolarization might lead to a persistent increase in protein kinase activation, oncogene activity, and other plasticity changes in the CNS [85,87]. Moreover, PIP cycle modulation has been found to regulate several membrane transport proteins (e.g., voltage-gated K^+^ and Ca^2+^ channels, ion channels that mediate sensory and nociceptive responses, epithelial transport proteins, and ionic exchangers) [85,88]. Therefore, altered mIns levels might be partially responsible for the cellular ion dysregulation seen in many neurodegenerative conditions [89].

With respect to its brain distribution, mIns is predominantly localized in astrocytes (~6 mM [84]), and not in neuronal cells (less than 0.5 mM [84]) [70,78,84], suggesting that mIns can be stored and regulated in glial cells before their utilization in the PIP cycle [84]. During glial proliferation, mIns levels have been shown to rise, making mIns a useful glial marker [25,79,84]. Elevated mIns has been observed in almost all studies of MS patients [78], not only in the active MS phase [25,90], but also in the non-active MS phase [52,91], and even in the early stages of the disease [24,92]. Elevated mIns has been observed in NAWM of RRMS, SPMS, and PPMS patients [51,54,77], in NAWM of CIS patients [78,93,94], in the cortical GM area of RRMS patients [51], and in the thalamus and hippocampus in RRMS, SPMS, and PPMS patients [56]. Higher mIns/tNAA has been found not only in the hypothalamus of patients with RRMS and SPMS [52], but also in early MS stages [24], suggesting that reactive gliosis appears from early to progressive MS manifestation. Furthermore, the same studies have determined a positive correlation of hypothalamic mIns/tNAA and patient disability, even in the early MS stage. In another study focusing on NAWM, mIns/NAA in WM has been suggested as a predictor of clinical disability in progressive MS [67,75]. In general, increased mIns in NAWM has been reported to precede axonal damage in MS [58,77,95], although the type and location of an MS lesion might influence the level of the immune response [9,22,25]. A typical, less prominent influx of immune cells attributable to the blood–brain barrier leakage has been detected in GM lesions compared with WM lesions [82,96,97]. However, deep GM lesions (in all MS stages) are associated with perivascular and parenchymal lymphocytic infiltration and are generally more sensitive with regard to inflammatory CNS reactions than those in the cortex [9]. Nevertheless, several studies have established that the neuro-axonal damage in WM might be at least partially reversible [8,51,93]. This is in agreement with the theory of the dual role of astrocytes that both contribute to axon demyelination and support their remyelination [58,98,99]. This means that an early glial response might inhibit cell death pathways via the triggering of neurotrophic factor expression [8,58]. Finally, given the predominant source of mIns is the PIP cycle, its astrocytic receptors are possible target regulators for the therapeutic action of mIns, although its exact involvement in MS progression is still not clear [84]. Some evidence suggests that the mIns level in the brain is associated with changes in mood state [83]. Administration of mIns has been found to be therapeutic for obsessive–compulsive and panic disorders [83,84], depression [84,85], bipolar disorders, and concomitant sleep symptoms [100,101].

#### 3.1.3. Neurotransmitter Dysregulation–Glx Excitotoxicity

**Figure 4:**
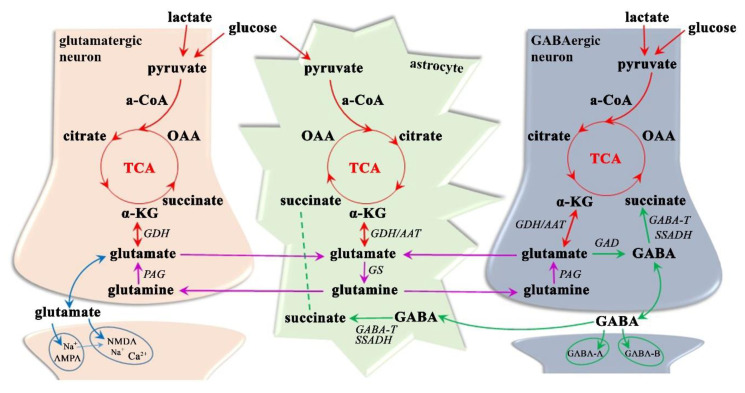
Schematic representation of glutamate-glutamine and γ-aminobutyric acid (GABA) metabolism. Glucose (optionally lactate) metabolism via pyruvate and acetyl coenzyme A (a-CoA) in neurons and astrocytes leads to the formation of citrate in the tricarboxylic acid (TCA) cycle resulting in the re-formation of oxaloacetate (OAA), ready for another turn of the cycle with a large amount of energy production. Citrate can be metabolized in the TCA cycle to α-ketoglutarate (α-KG), which can leave the cycle to form glutamate catalyzed by aspartate aminotransferase (AAT) or glutamate dehydrogenase (GDH). Glutamate can be combined with ammonia to produce glutamine via glutamine synthase (GS) in astrocytes and transported to neurons (both glutamatergic and GABAergic) where it forms glutamate via phosphate-activated glutaminase (PAG). After its transportation back to the astrocytes, the glutamate-glutamine cycle is complete. In glutamatergic neurons, glutamate can also be accumulated in vesicles and released as a neurotransmitter. Most glutamatergic activity stimulates α-amino-3-hydroxy-5-methyl-4-isoxazole propionic acid (AMPA) receptors, which are permeable to Na^+^ ions. Under persistent stimulation, the voltage-dependent bound Mg^2+^ ion is removed from the N-methyl-D-aspartate (NMDA) receptor enabling an influx of both Na^+^ and Ca^2+^. However, cytosolic glutamate might be reuptaken by glutamatergic neurons and reconverted to aspartate during the transamination of glutamate to α-KG catalyzed by glutamate dehydrogenase (GDH). Metabolism of α-KG is linked to the TCA cycle. In addition, glutamate is the main precursor of GABA, whose synthesis is based on glutamate decarboxylase (GAD) in GABAergic neurons. In neurons and astrocytes, GABA can be transaminated via GABA-transaminase (GABA-T) to succinic-semialdehyde and further oxidized by succinate semialdehyde dehydrogenase (SSADH) to succinate, which is also an intermediate of the TCA cycle. Moreover, GABA is a major inhibitory neurotransmitter that influences both ionotropic GABA-A and metabotropic GABA-B receptors, causing the inhibition of neurotransmission.

Despite glutamate not crossing the blood–brain barrier [102,103,104], it shows appreciable concentrations throughout the CNS (5–10 mM [104]), suggesting that it has metabolic importance. Although it can be found in all brain cells, its highest levels are reached in the synaptic vesicles of nerve terminals, from where it can be released into the synaptic cleft [104,105]. Glutamate is physiologically taken back up into neurons and astrocytes but can be synthesized (Figure 4) from glucose or pyruvate (through the TCA cycle and α-ketoglutarate, which receives an amino group by the activity of aminotransferase in astrocytes) or from glutamine (by phosphate-activated glutaminase in neurons), in addition to being formed from α-ketoglutarate (by glutamate dehydrogenase in astrocytes or neurons) [87,102,106]. Given that the glutamate pool is maintained in equilibrium, it is predominantly accumulated in neuronal synaptic vesicles (based on vesicular glutamate transporters-1, 2, and 3) but can also be reconverted back to α-ketoglutarate and metabolized through the TCA cycle in both astroglia and neurons and transformed to GABA (by glutamate decarboxylase in GABAergic neurons) or glutamine (by glutamine synthetase in astrocytes) [103,105,106]. In particular, the glutamate-glutamine cycle between neurons and astroglia is highly active, representing approximately 85% of the glutamate turnover rate [106]. This neuroglial transmitter recycling is an essential transporting system to ensure not only the astrocytic release of glutamine (Na^+^-dependent neutral amino acid transporters-3 and 5) and its neural uptake (Na^+^-dependent neutral amino acid transporters-1,2, and 7; slc38-family; alanine-serine-cysteine transporter-2) [87,105], but also the neuronal leakage of glutamate (vesicular exocytosis; anion channels; reversed glutamate transport proteins, glutamate/cystine exchanger) and its ingestion into astrocytes (glutamate transporter-1; glutamate aspartate transporter; excitatory amino acid transporters-1 and 2) and neurons (Excitatory amino acid transporter-3, ionotropic and metabotropic glutamate receptors) [87,104,105].

Glutamate has a myriad of functions in the CNS with key roles in protein synthesis, ammonia fixation, and nitrogen and energy metabolism, and in the establishment of the complex communication networks (synapse formation, dendrite pruning, cell migration, and differentiation) between neurons, astrocytes, oligodendrocytes, and endothelial and immune cells [20,107]. Taking into account that approximately 85% of cortical neurons are glutamatergic [12,103], glutamate is considered as the most important excitatory neurotransmitter; it is involved in the regulation of circadian rhythms, sensory-motoric coordination, and emotional and cognitional functions, including memory formation and retrieval [11,103]. Typical glutamate (Figure 4) post-synaptic activity influences three types of ionotropic glutamate receptors, namely α-amino-3-hydroxy-5-methyl-4-isoxazole propionic acid (AMPA), N-methyl-D-aspartate (NMDA), and kainate receptors [87,105]. Whereas AMPA is permeable to Na^+^ ions, its activation causes the depolarization of the postsynaptic membrane and is thought to be responsible for the majority of fast glutamate-mediated neurotransmission [87,104]. Depolarization might remove the voltage-dependent Mg^2+^ ion bound from the NMDA receptor, allowing an influx of both Na^+^ and Ca^2+^ to the dendrite and enabling activation during high-frequency stimulation (i.e., memory formation, synaptic plasticity) [21,104]. The numerous triggers, such as energy depletion and mitochondrial dysfunction [8,20,107], decrease the activity of mitochondrial respiratory chain complexes I and III [8], calcium overload [107], oxidative stress [21], and higher microvascular permeability [108], activate microglia, dendritic cells, and macrophages [21,107], and upregulate glutamate transporters and receptors causing a disturbance in glutamate homeostasis that leads to neural hyperexcitability [20,21,108]. Glutamate excitotoxicity is principally mediated by the excessive influx of Ca^2+^ via NMDA receptors [102,104,105] and leads to multiple adverse effects including the impairment of intracellular Ca^2+^ homeostasis, the dysfunction of mitochondria and endoplasmic reticulum, an increase in free radicals, the persistent activation of phospholipases, proteases, endonucleases, and kinases, and an increase in the expression of pro-death transcription factors and immediate early genes ultimately leading to neuronal and glial cell apoptosis [13,50,109]. Microglial activation and subsequent reactive gliosis both precede and follow the final stages of neurodegeneration caused by glutamate excitotoxicity [21,108].

Recently developed radiological and biochemical methods have provided evidence that excessive activation of the glutamatergic pathway plays an important role in MS pathophysiology [20,107,108]. Since previous research has shown that glutamate occurs in the brain at approximately 10-times higher concentrations than glutamine [110], the Glx peak (Figure 1) is thought to be primarily driven by the glutamate signal. Furthermore, the glutamate level has been found to be elevated, whereas the glutamine concentration persists unchanged in the MS brain [51,90]. Elevated Glx, as measured by MRS, has been reported in various CNS structures: in NAWM and in active (but not in chronic) WM lesions of RRMS [70,111], SPMS, and PPMS patients [77], and in NAWM and the thalamus in CDMS patients [51]. Therefore, glutamate has been proposed as a predictive marker for WM pathology [51,70]. The findings in GM are contradictory: Glx is elevated in cortical GM and hypothalamus in RRMS and SPMS [52,70], is unchanged in the thalamus, hippocampus, and cortical GM in RRMS, SPMS, and PPMS [56], or declines in cortical GM [1] and in the hippocampus, thalamus, cingulate, and parietal cortices in RRMS [111]. Altered Glx metabolism in the hypothalamus has previously been shown, in RRMS and SPMS, to correlate with disease severity [52,58] and in the early MS state suggesting a positive correlation to disability in MS patients [24]. This indicates that Glx in the hypothalamus of RRMS/SPMS patients is associated with MS manifestations (disease duration, depression, fatigue), even when no MR-visible lesions are detectable in the GM [52]. Elevated glutamate has further been demonstrated to lead to the destruction of oligodendrocytes, a characteristic that is directly related to disease progression [112,113]. The increase in glutamatergic transmission observed in the GM of MS patients has been clearly shown to result in cognitive impairments, even in the early phase of MS pathogenesis before the appearance of severe motor impairments [24,108]. In summary, Glx has the potential to be used as a biomarker of the progression and severity of MS and should be evaluated in WM and GM brain areas [21,24,58].

#### 3.1.4. Neurotransmitter Dysregulation–GABA Inhibition

Because 25–45% of neurons of the CNS are GABAergic [12,114], GABA is considered as a major inhibitory neurotransmitter that is essential for neuronal plasticity, i.e., for the formation of action potentials during learning and memory formation, offensive-defensive behavior and sensory-motoric control [11,23]. Its physiological levels inhibit neuronal hyper-excitability that can manifest as irritability, seizures, headaches, movement disorders, tics, epilepsy, anxiety, insomnia, fatigue, or psychiatric disorders such as depression and focus and attention difficulties [12,13]. However, excessive levels of GABAergic activity can cause sedation, sleepiness, and lethargy [11,115].

The maintenance of suitable GABA levels is dependent on the GABA reuptake rate by transporter proteins (GABA transporter-1 in presynaptic nerve terminals, GABA transporter-3 in glial cells) and on the ability of the brain to form glutamate as the main precursor of GABA synthesis and to degrade GABA via the TCA cycle [23,87]. For GABA synthesis (Figure 4), the essential glutamate decarboxylase enzyme is expressed solely in GABAergic neurons, whereas key roles during GABA catabolism are played by the mitochondrial enzymes GABA-transaminase and succinate semialdehyde dehydrogenase, which are localized in astroglial and neural cells [13,23,87]. In both enzymatic processes, mitochondria are critical and sensitive to changes in nutrient and oxygen supplementation and to the loss of the trophic support of incoming and outgoing fibers, as occurs in the brain of MS patients [8,16,116]. GABA transmission (Figure 4) itself is based on Ca^2+^-dependent GABA release during the depolarization of the presynaptic membrane and on ionotropic GABA-A (activates the rapid and transient opening of Cl^−^ channels) and metabotropic GABA-B (activates, via coupled G-protein, inwardly rectifying K^+^ channels and inhibits voltage-activated Ca^2+^ channels) receptors causing the hyperpolarization of the neuron and the inhibition of neurotransmission [87,117]. Since both types of receptors have been found not only post- but also pre-synaptically on GABAergic neurons, GABA is thought to regulate their release via voltage-activated Ca^2+^ channels [117,118].

In MS, the brain has been demonstrated to exhibit both reduced GABA-related gene transcripts [8] and a decline in the density of GABAergic neurons or GABA receptors [119,120,121]. This might lead to a topographic variation in GABA levels (Figure 1) in patients with progressive MS, with decreases in key brain regions, including the posterior cingulate cortex (containing WM and GM) [121], the sensorimotor cortex, and the hippocampus [23,119,122]. Interestingly, inflammation rather than demyelination, being present in both active and inactive MS lesion types, is thought to be involved in the regulation of GABA transmission [120]. A decline in GABA inhibition might result in higher energetic demands and, in the case of energetic misbalance (i.e., insufficient adenosine triphosphate (ATP) production by mitochondria, a reduction in respiratory chain complexes I and III), might cause the degeneration of chronically demyelinated axons in MS, as is supported by the disruption in ion homeostasis (impaired voltage-gated ion channels, Na^+^/K^+^-ATPase, Na^+^/Ca^2+^ exchanger) along the demyelinated axolemma [8,23]. These processes might further lead to neuroaxonal degeneration and, finally, the loss of compensatory mechanisms that maintain physiological functions [23,72]. In support of this assumption, a worsening of MS manifestations associated with GABA decrements has been observed in several studies, i.e., lower GABA in the hippocampus and the sensorimotor cortex has been correlated with a decrease in grip and muscle strength [23] and reduced motor performance [121]; in SPMS; GABA decrements in the sensorimotor cortex have been associated with the deterioration of fine-motor coordination in RRMS [119]; decreased hippocampal GABA has been connected to distinct memory deficits in RRMS [122]; and reduced GABA in the posterior cingulate cortex and hippocampus has been associated with cognitive impairment in RRMS [123]. Furthermore, a decreased GABA concentration in the hippocampus and the sensorimotor cortex of SPMS patients has been proposed as a predictive marker of physical disability in MS progression [23]. These reports suggest that GABA modulation and neurotransmission represent a critical neuroprotective approach in MS management. Moreover, based on animal studies, selective GABA-A receptor agonists or positive allosteric modulators have been established to increase the GABA concentration and to block Ca^2+^ channels, thereby enhancing the potential of this methodology for MS treatment [117].

#### 3.1.5. Limitations of ^1^H MRS

Despite the evidence that ^1^H MRS reveals numerous affected biomarkers for the evaluation of MS progression, this radiological technique is still not routinely used as MS diagnostic tool. The reason is apparent, namely missing unambiguous predictive or distinguishable biomarkers. Furthermore, some technical weaknesses of this method are still awaiting fine-tuning. One persisting ^1^H MRS issue is the presence of an unwanted signal, namely the strong signal of water and subcutaneous lipids or signal contamination from adjacent tissue. Since the abundant cerebellar water signal (~80 M) covers the peaks of other metabolites (~10 mM), techniques such as chemical shift selective water suppression, variable pulse power, and optimized relaxation delays, and relaxation time manipulations are routinely applied to suppress its peak [26,37,124]. Analytical software such as LCModel can also distinguish and eliminate the residual water signal [37,125]. Furthermore, pericranial fat contains high concentrations of lipids that can also strongly contaminate ^1^H MRS [26,37]. Similarly, the proximity of bone structures and air-tissue interfaces (e.g., the paranasal sinuses, inferior and anterior temporal cortices, orbitofrontal regions) can result in spectral line broadening and homogeneity and susceptibility artifacts [37,124]. Therefore, the positioning of measured voxels away from these unwanted areas is essential. Furthermore, the application of outer-volume suppression also helps to avoid signal contamination [37,124]. The potential pitfall of CSI is the occurrence of signal spreading from adjacent voxels (the so-called point-of-spread function or voxel bleeding), which is commonly reduced by k-space filtering methods [124]. Another technical issue is the quantification of the absolute metabolite concentration in ^1^H MRS. The integral of tCr is widely used to evaluate relative metabolites ratios [37,41]; nevertheless, inconsistencies in the reported increased, decreased, and absent tCr alternations in the lesions and NAWM of MS patients are apparent [25,51]. Therefore, one technique increasingly being used for absolute metabolite quantification employs the water peak as a standard of known concentration [3,26]. Another challenge is the evaluation of several small molecules that, by standard ^1^H MRS examination, are difficult to distinguish in MR spectra, although they might be clinically useful as diagnostic biomarkers. This is the case for GABA, whose accurate quantification is complicated because of the interaction between adjacent nuclei in the same molecule (*J*-coupling) causing multiplets (at 1.88, 2.28, and 3.02 ppm) and the overlapping by other metabolite resonances (tCr, Glx, tNAA, and macromolecules) [38,39]. Thus, several MRS-editing techniques have been evaluated for GABA quantification (e.g., Mescher–Garwood editing [38,39], 2D localized correlation spectroscopy [121], homonuclear J-difference editing, and multiple quantum filtering [12]). Likewise, the separation of glutamate and glutamine is challenging because of their structural similarities and *J*-coupling interactions [11,115]; however, several specific MR sequences are being developed specifically for this task (e.g., the spectrally selective refocusing method [126] and the point-resolved spectroscopy asymmetry method [127]). Obviously, higher field (≥3 T) MR scanners offer a higher spectral quality and spatial resolution (a higher signal-to-noise-ratio, decreased transverse relaxation times and increased magnetic susceptibility effects) but are also somewhat limited (e.g., localization and phase artifacts, chemical shift errors, and B_1_ inhomogeneities) [38]. The remaining challenge is the whole-brain coverage during one investigative procedure, which is highly desirable in most clinical applications. The use of SVS is possible to measure volumes within the 3–8 cm^3^ voxel size [37,125,128], whereas CSI enables the minimalization of the individual voxel size to 0.5–1.5 cm^3^ [128,129,130] in one or several slices (single-slice two-dimensional (2D) CSI; multi-slice three-dimensional (3D) CSI) [37,125,128]. Furthermore, whole-brain CSI sequences based on time-sufficient echo-planar CSI [37,129,131], parallel- or spiral-encoded CSI [37,129], or free induction decay CSI [131] have been achieved; however, these methods are also still limited.

### 3.2. ^31^P MRS

In general, ^31^P MRS has excellent potential for clinical neurological practice (Table 2) because of its noninvasive in vivo assessment of cellular energy metabolism and the indirect evaluation of the phospholipid composition of the cell membrane, intracellular pH, and intracellular Mg^2+^ concentration [132,133,134].

#### 3.2.1. Phospholipids Metabolism

The synaptic strength and plasticity of neuronal networks are dependent upon the composition and properties of cell membranes comprising a phospholipid double-layer with immersed proteins responsible for cellular membrane fluidity and permeability, intracellular homeostasis, actin cytoskeleton maintenance, channel and receptor formation, signal transduction, and the regulation of various enzymatic reactions including cellular metabolism, proliferation, and cell death [132,133,134,135].

**Figure 5:**
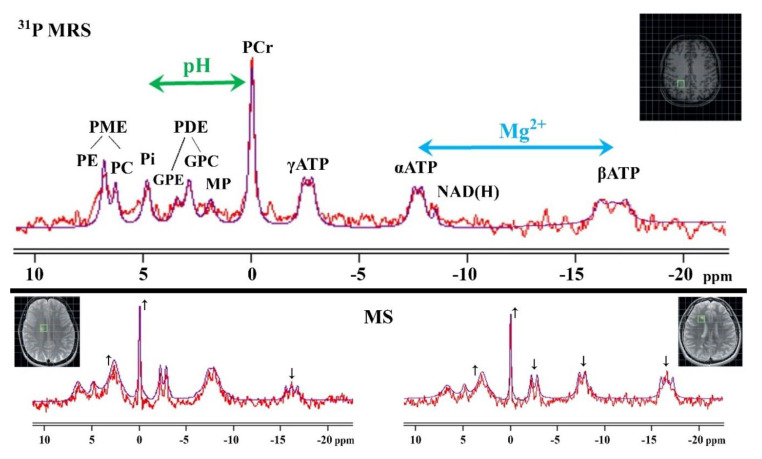
Visualization of phosphorus-31 magnetic resonance spectroscopy (^31^H MRS). ^31^P MRS brain tissue examination represents the measured signal from the voxel depicted on MRI in green square. The following metabolite peaks are shown: phosphoethanolamine (PE), phosphocholine (PC), phosphomonoesters (PME), inorganic phosphate (Pi), glycerophosphoethanolamine (GPE), glycerophosphocholine (GPC), phosphodiesters (PDE), membrane phospholipids (MP), phosphocreatine (PCr), nicotinamide adenine dinucleotide (NAD(H)), and adenosine triphosphates (α-, β-, γ-ATP). Graphical representations of the indirect pH (chemical shift of Pi and PCr depicted as green arrow) and [Mg^2+^] evaluation (chemical shift of α-ATP and β-ATP depicted as blue arrow) are also indicated in the fitted MR spectra. The ^31^P MRS spectra for a healthy (at the top) and MS-affected (at the bottom) brain are also depicted together with the highlight of the typical metabolic peaks changes via small black arrows (↓: decreased, ↑: increased).

Although the ^31^P MRS (Figure 5) does not enable the direct measurement of membrane phospholipids because of their fixed membrane integration [41,135], it is suitable (even at lower magnetic fields such as 1.5T) for detecting phospholipid precursors, namely phosphomonoesters (PME, i.e., phosphocholine, phosphoethanolamine), and their degradation products, namely phosphodiesters (PDE, i.e., glycerophosphocholine, glycerophosphoethanolamine) [132,133,134]. The separation of peak sub-contributor signals (at >3T) increases the diagnostic accuracy of a ^31^P MRS examination [132,133,136]. Nevertheless, increased PME might suggest membrane proliferation, whereas increased PDE might reflect cell membrane breakdown [134,137,138]. Moreover, the metabolic ratio of PME/PDE is generally considered to be an appropriate indicator of cell membrane turnover and to be representative of changes in the phospholipid double layer [139,140]. Both PME and PDE (slightly PDE > PME) peaks are detectable physiologically in WM and in GM (and are generally higher in WM) [140,141], with a gradual increase in PDE and a reduction in PME during aging, probably reflecting the cell membrane condition [142,143,144]. In the acute MS lesions of CDMS, PPMS, and SPMS patients, increased PDE concentrations (mainly attributable to the glycerophosphocholine) have been found [145,146], confirming higher myelin phospholipid degradation. Discrepancies have been perceived in the NAWM of MS patients, showing not only increased PDE levels in CDMS patients [147], but also decreased levels in SPMS and RRMS patients [137,148], supporting the hypothesis of the impact of lesion localization and of neuro-axonal tracking failure in MS patients. Moreover, a higher PME and increased PME/PDE ratio have previously been suggested to result from reactive astrogliosis [137]. Astrocytes actively participate in MS progression by means of their ability to inhibit remyelination and axonal regeneration not only by forming a glial scar, but also by supporting MS lesion formation via the production of cytotoxic factors and by contributing to mitochondrial dysfunction [98,99,137]. Therefore, an increased PME/PDE ratio is thought to provide an early indication of both demyelination and remyelination in MS patients [140,146].

#### 3.2.2. Cellular Energy Metabolism

Cellular energy metabolism is crucial for most cellular processes and functions, including the maintenance of ionic gradients (mainly NA^+^/K^+^-ATPase), the activation of protein and lipid biosynthesis, and phosphorylation, cell proliferation, neurotransmitter metabolism, and transport, plus signal transduction [135,137,138]. Therefore, ^31^P MRS (Figure 5) is regarded as a useful clinical tool for the noninvasive evaluation of brain tissue energetic processes by means of peaks of adenosine triphosphate (ATP, i.e., α-, β-, γ-ATP), phosphocreatine (PCr), and inorganic phosphate (Pi) [132,149,150]. Their metabolic pathways are tightly coupled via the enzyme creatine kinase-B (CK-B; the cytosolic isoform expressed highly in astrocytes, but to a lower extent in neurons [116,151]), which generates ATP by the transfer of a phosphate Pi group from PCr to adenosine diphosphate (ADP) under conditions of higher energy demands and/or insufficient ATP production through cytosolic glycolysis and mitochondrial oxidative phosphorylation [16,73,132]. The result of such a transfer is the decline of cellular PCr and the relative increase in ATP and Pi [137]. However, during ATP hydrolysis (via the mitochondrial CK-B isoform distributed in glial and neural cells [116,151]), PCr and ADP are formed, and the energy from the high-energy phosphoanhydridic bonds is released [41,149,152]. In this regard, ATP is considered as a biomarker of the momentary energetic state of the cell [134,153].

Under physiological conditions in brain cells, the majority of glucose is metabolized oxidatively, leading to a steady PCr-ATP-Pi (stable concentration ~3 mM ATP, ~4 mM PCr, ~1 mM Pi [154]) chemical exchange system [135,152,155]. In particular, PCr is considered as a stable compound in healthy brain tissue. However, higher PCr levels have been found in GM compared with WM [132,142], showing physiologically higher ATP consumption in GM. Nevertheless, PCr is usually taken as a reference for relative MRS quantification [134,141,142], although it is rapidly utilized for fast ATP recovery in the case of higher energetic demands [142,149,153]. In these cases, the mitochondrial compensation mechanisms physiologically increase the effectiveness and/or amount of CK-B for the maintenance of cellular energetic stability [16,142]. Nevertheless, in a postmortem study of progressive MS subjects, a reduced count and activity of CK-B was found in WM astrocytes, suggesting the probable cause of the disrupted PCr levels in NAWM of these patients [16]. Changes in CK-B throughout the cerebral WM of MS patients has been defined both as reduced transcription and as post-translational enzyme modification, probably because of the increased production of reactive oxygen species in MS [137,156]. In agreement with this finding, dysfunctional astrocytes in MS have previously been stated to influence axonal mitochondria causing their energetic failure, and thus they might contribute to axonal degeneration [16,116,156]. In general, PCr is considered as a sensitive marker for mitochondrial rather than for CK-B or astrocytic efficiency, because its increase usually reflects mitochondrial dysfunction and/or tissue excessive requirements [134,137,138]. Indeed, mitochondria contain the energy-dependent respiratory chain [8,157]. In several MS studies, PCr has been found to be elevated in acute MS lesions [137,148] and in NAWM of RRMS, SPMS, and PPMS patients [16,137,147], suggesting the low metabolic state of the brain tissue, even before WM lesions are formed. Furthermore, the increase in PCr has been established to be correlated with the severity of disability and is higher in patients with a more progressive course [148]. Similarly, the NAWM of RRMS and SPMS patients exhibits a negative correlation of β-ATP and disease severity [137], suggesting that a more progressed disease involves lower ATP production.

The inflammatory environment in MS has also been suggested to cause a decrease in mitochondrial activity and ATP production [158]. Based on the disrupted NA^+^/K^+^-ATPase in the cells, intracellular Na^+^ accumulation followed by the reversed activity of NA^+^/Ca^2+^ transporters potentially leads to increasing Ca^2+^ and glutamate concentrations in cells, with neuro-axonal degenerative consequences [73,137]. Moreover, in the NAWM of RRMS patients, a higher PCr/β-ATP level has been found compared with that in SPMS patients [156]. These observations suggest a defective energetic metabolism in MS and a higher compensatory mechanism during the remyelination phase of the disease [137,150]. Finally, the β-ATP level and PCr/β-ATP ratio have been suggested as candidates for biomarkers in the assessment of MS disease severity [116,137,156]. The indicated putative mitochondrial disorders in MS manifestation indicate that a therapeutic approach focusing on mitochondria impairment (e.g., biotin, prucalopride, fluoxetine) will probably play an important role in MS treatment; however, their effects are still under consideration [73,94,146].

#### 3.2.3. Intracellular pH

Intracellular pH can be evaluated non-invasively by ^31^P MRS (Figure 5) based on the chemical shift of endogenous Pi from the PCr peak [134,154,159]. The Pi position is determined by its form of a conjugated pair of anions, namely H_2_PO_4_^−^ and HPO_4_^2−^, which change rapidly depending on the relevant dissociation reactions [160,161]. The following formula is used for pH evaluation:

(1) $$pH=\mathrm{pKa}+\log\frac{\delta_{Pi}-\delta_{a}}{\delta_{b}-\;\delta_{Pi}}$$

where the anion deprotonation constant (pKa = 6.73) and the ^31^P-limiting shifts for H_2_PO_4_^−^ (*δa* = 3.275 ppm) and HPO_4_^2−^ (*δb* = 5.685 ppm) are used in the data analysis [154]. The modulation of human brain pH is achieved by osmotic and metabolic regulatory actions that are related to the transport and diffusion of ions, buffer systems, the activity of carbonic anhydrase, and energy consumption [132,159,162]. Several studies have reported intracellular pH levels in the healthy human brain in the range of 7.01–7.07 [161,163], with (approximately) a 0.5% decrement per decade [142]. A relatively stable intracellular pH in brain tissue is important because its change can affect several cellular processes (i.e., cellular enzyme activity, ion channel/transporter efficiency) and thus have an impact on many cell functions (e.g., neuronal activity, synaptic transmissions, mitochondrial functions, pathway regulation) ultimately leading to cell life or death [135,155,159]. In acute MS, lesions have been found with unchanged or slightly alkaline [145,146,148] intracellular pH values compared with healthy brain tissue, suggesting the occurrence of various metabolic processes in the observed brain tissue.

#### 3.2.4. Intracellular Mg^2+^

The indirect ^31^P MRS measurement of free cytosolic Mg^2+^ is based on its correlation to the chemical shift of the β-ATP signal from α-ATP (Figure 5), taking into account that most cytosolic phosphate compounds bind Mg^2+^ [132,154,164]. For [Mg^2+^] evaluation, this is mostly applied in the following formula:

(2) $$\left[Mg^{2+}\right]=k_{d}\frac{\delta_{ATP}-\delta_{\alpha -\beta }}{\delta_{\alpha -\beta }-\delta_{MgATP}}$$

where the *MgATP* effective disassociation constant (*k_d_* = 0.05 mM) and the limiting shifts for ATP (*δ_ATP_* = 10.82 ppm), *MgATP* (*δMgATP* = 8.32 ppm), and α-, β-ATP (measured *δ*_α−β_ in ppm) are used in the data analysis [154]. The noninvasive examination of the in vivo intracellular [Mg^2+^] in the CNS provides a unique possibility for assessing Mg^2+^ homeostasis and its implementation in brain pathophysiological processes.

Magnesium is involved in a myriad of biochemical processes including the activation of more than 300 enzymes [165,166,167] and, thus, is essential for the regulation of ion channels and signaling pathways [95,168], the synthesis of proteins, macromolecules, and DNA, oxidative phosphorylation, cell membrane remodeling, and stabilization, cell growth and proliferation, and immune responses [95,164,169]. Although approximately 24 Mg^2+^ transport mechanisms and homeostatic factors have been identified [168,170]), the most essential eukaryotic homologs of the Mg^2+^ transporter are widely accepted to be CorA–Mrs2 proteins operating in the inner mitochondrial membrane [95]. Furthermore, mitochondria have been established as the cellular organelles with the highest [Mg^2+^] (0.2–1.5 mM) [95,164]. Therefore, mitochondria are considered to be the main sites of storage of cellular Mg^2+^ [95,109]. On this basis, the evaluation of intracellular [Mg^2+^] has been suggested to be particularly important in neurological diseases in which energetic mitochondrial dysfunction is probably the primary causative or putative pathogenic factor (including mitochondrial cytopathies, migraines, multiple system failures such as MS, Parkinson’s disease, etc.) [164,171]. However, the mechanisms of Mg^2+^ decline in pathological conditions and the availability of this ion in the neural tissue after its administration are still unclear [170,172]. Nevertheless, magnesium therapy has been successfully utilized in the treatment of symptoms that usually occur in MS, including depression [173], anxiety [174], sleep disorders, insomnia, chronic fatigue [175], pain [176], and constipation [177]. In experimental traumatic brain injuries in rats, a decline has been observed in intracellular [Mg^2+^], correlating with motor deficits [178] that show a significant improvement after magnesium supplementation [179].

Magnesium has been established to reduce brain edema and cerebral vasospasms, to restore blood–brain barrier effectiveness [109,180], and to ensure neuro-axonal protection against nitric oxide and superoxide radicals attributable to the direct inhibition of nitric oxide synthase by Mg^2+^ [167]. These radicals might promote oligodendrocyte injury, demyelination, and axonal damage caused by the inhibition of mitochondrial respiration and intra-axonal Na^+^ and Ca^2+^ accumulation [109,181]. Further protective Mg^2+^ mechanisms might include the inhibition of the release and uptake of glutamate through the NMDA receptor and the blockade of Ca^2+^ channels [182,183], causing the hyper-excitability of neurons and leading to cell death [109,184]. Extracellular Mg^2+^ deficiency might also induce the apoptotic process, mainly through increased oxidative stress (i.e., inflammation, cytokine and phagocyte activation and oxidative DNA damage [109,185]), accompanied by the intracellular Mg^2+^ mobilization from mitochondrial stores that is necessary for stimulating the activity of Ca^2+^/Mg^2+^-dependent endonucleases acting in nucleosomal DNA fragmentation [109]. From this point of view, magnesium supplementation should, in principle, be considered as an anti-apoptotic factor [182,183]. This is supported by the evidently better clinical condition of RRMS patients who attain a healthy Mg serum content after dietary supplementation compared with patients with MS-typical serologically inadequate Mg levels [166]. Alternations in the brain [Mg^2+^] might be regarded as a possible mechanism contributing to synaptic strength or neuronal plasticity (with no WM-GM differences in magnesium content [132]). Consequently, they might alter memory capacities or affect cognitive ability during aging [165]. A decline in brain [Mg^2+^] in traumatic brain injury has been established to be associated with the development of cognitive deficits [109]. In MS progression, an inverse association has been confirmed between Mg^2+^ intake and tissue damage throughout WM lesions [167]. This is in agreement with a postmortem study focusing on CNS in MS patients showing a significant decline in [Mg^2+^], with the most marked reduction occurring in demyelinated WM plaques [186]. In principle, abnormal [Mg^2+^] in MS patients should be monitored (e.g., serum level, ^31^P MRS in the brain) and eventually corrected, as this may at least moderate MS manifestations [166].

#### 3.2.5. Limitations of ^31^P MRS

Although ^31^P MRS is a modern noninvasive MR technique providing useful in vivo assessment of pathophysiological brain conditions, it is currently not accepted as a diagnostic tool in clinical practice. One reason is the gyromagnetic ratio (a physical characteristic expressing the interaction between nuclei and magnetic field) of ^31^P nuclei, which is approximately 2.5 times lower than for ^1^H, resulting in lower resonance frequency and much lower detection sensitivity [125,132]. These factors imply that, in order to obtain a satisfactory ^31^P spectrum, it is necessary to apply special MR equipment (special radiofrequency channel, additional radiofrequency coils) able to work at the resonance frequency of the ^31^P nuclei [118,120,148]. However, with progressive technical and software improvements, ^31^P MRS has become more easily implemented [140,162]. Moreover, because of the short transverse relaxation time of the ^31^P metabolites, it is not advisable to use common in-built scanner sequences for ^1^H MRS such as stimulated echo acquisition mode (STEAM) and point-resolved spectroscopy (PRESS), but it is recommended to apply rarely implemented techniques such as image-selected in vivo spectroscopy (ISIS) or pulse acquire techniques [26,132,161]. Furthermore, most of the ^31^P MRS metabolites are in a spectral curve presented as double, triple, or multiple peaks because of *J*-coupling, which needs to be correctly fitted or, even better, reduced by decoupling techniques [132,141]. Another possible reason for this method not being considered in the medical field is that its applicability, chiefly on lower magnetic field MR scanners (<3T), is limited by the insufficient spectral resolution and its lower signal-to-noise-ratio [134,138], resulting in relatively long acquisition times or larger measurement volumes (typically ~ 40–100 cc; rarely ~ 20 cc [132,162]). Patients do not ideally tolerate such drawbacks [136,161]. Furthermore, more substantial volumes, in turn, make it a challenge to study tissue heterogeneity (e.g., MS lesions). Generally, ^31^P MRS examinations under higher magnetic fields (ideally 3T or 7T) provide a better spectral resolution, higher repeatability, and signal improvements that enable the measurement of an extended range of biomarkers (i.e., phosphocholine, phosphoethanolamine; glycerophosphocholine, glycerophosphoethanolamine; Figure 5), and the examination of smaller volumes and/or the use of shorter data collection times to monitor dynamic disease within one integrative study [132,134,136].

## 4. Parametric T_2_-Relaxation Mapping

**Figure 6:**
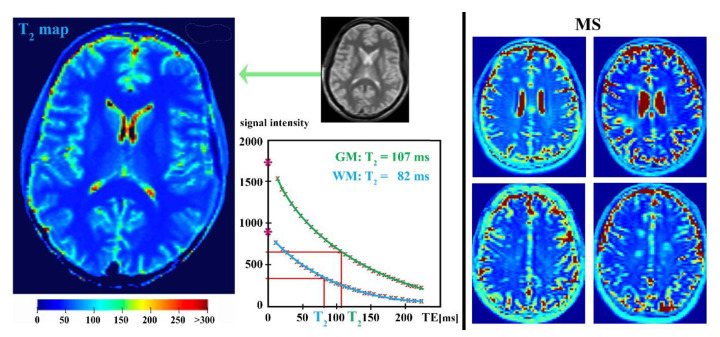
Visualization of the T_2_ relaxation time mapping. T_2_ relaxation time mapping of the brain tissue showing measured relaxation signal decays (relaxation curves, blue for white matter (WM) and green for gray matter (GM)), including relaxation T_2_ time values for both WM and GM. The T_2_ parameter maps for healthy (on the left) and MS-affected (on the right) brain are also depicted.

T_2_-weighted MRIs are often used as a supporting clinical tool for the diagnosis of various brain pathologies, including MS [28,29,33,41]. The distinguishing of brain tissue alterations on these scans is based on the different T_2_ relaxation times (Figure 6) that reflect the exchange of energy between spins on a molecular scale [26,187]. The widely used T_2_ relaxometry method is the multiple spin echo sequence (usually the Carr–Purcell–Meiboom–Gill sequence) exploiting the phase coherence of nuclear spins after a 90° radiofrequency pulse followed by a series of 180° refocusing pulses [188,189]. This arrangement is gradually lost because of the spin–spin interactions and magnetic field inhomogeneities, which manifest as the gradual decay of the transverse magnetization [187,188,190]. For T_2_ relaxation measurement, single/multi-slice images that are repeatedly collected at several echo times are usually suitable, enabling the exponential loss of the signal to be fitted at each image pixel [188,189,190]. Thus, T_2_ is the time required for the transverse magnetization to fall to approximately 37% of its initial value [188,189].

### 4.1. Myelin Water Mapping

Substantially longer T_2_ relaxation times are exhibited by small, free, and fast-moving molecules, unlike the more efficient T_2_ relaxation of larger macromolecules with a stable molecular structure supporting the existence of strong local magnetic fields [191,192]. Several studies suggest that the T_2_ relaxation time is a potential biological parameter enabling the noninvasive in vivo evaluation of changes in tissue composition, chemical environment, or interactions [187,193,194]. In general, T_2_ relaxation times in healthy brain tissue (Figure 6) have been demonstrated to vary depending on the ratio of WM (~75 ms at 1.5T/~60 ms at 3T) to GM (~95 ms at 1.5T/~80 ms at 3T) or cerebrospinal fluid (~220 ms at 1.5T/~200 ms at 3T) [192,195,196]. The typical finding in MS, namely myelin loss, can be observed in T_2_-weighted MRI scans as hyperintense areas (lesions, plaques) that are characterized by prolonged T_2_ relaxation times [3,197,198]. The prolongation of T_2_ relaxation in MS lesions is thought to be caused by increased extracellular space and by larger cellular water intake in the less hydrophobic environment triggered by demyelination in WM [3,27]. Although prolonged T_2_ relaxation times have been found in several CNS regions, including the NAWM of MS patients [198,199,200], other research reports indicate that NAWM water T_2_ relaxation times are correlated with the tNAA concentration in RRMS patients [198,201], suggesting that an increase in lesion water reflects the degree of local demyelination, even before MS lesions are formed.

### 4.2. Iron Mapping Quantification

Several studies have shown that T_2_ relaxation is also affected (shortened T_2_ relaxation time, hypointensities on T_2_-weighted MRI) by iron accumulation [3,9,202]. In principle, MRI techniques are sensitive to local magnetic field variations caused by iron compounds that affect MR-relaxation parameters [203]. In the human brain, the following four main iron particles are found: hemoglobin-bound iron, which is present in the blood and which exhibits nearly paramagnetic behavior; magnetite- or maghemite-bound ion; iron bound to transferrin (the main iron transporter protein in the brain [204,205]) or ferroportin; and iron stores such as ferritin (the main intracellular iron storage protein in the brain [11,18,206]) or hemosiderin [203,207].

Cerebral iron ions released from oligodendrocytes and myelin fibers during demyelination is thus physiologically amplified in the healthy aging human brain [205,208,209]. Under physiological conditions, the elevated iron concentrations are found in the GM of the midbrain, especially in the basal ganglia [9,208]. Iron ions are required in the production of neurotransmitters to preserve the generation of signals [17,208]. Increased iron levels (attributable to age pathology) have been connected to the disruption of the brain iron homeostasis leading to neurodegeneration, including MS [9,202]. MS patients have further been shown to exhibit increased levels of soluble transferrin (main iron transporter protein in the brain [204,205]) receptor associated with reinforced iron turnover [10,205,210]. Another study has reported an increased level of ferritin in the cerebrospinal fluid and serum of SPMS patients [211], providing the possible reason for extra iron delivery, since the H-ferritin receptor in neurons and oligodendrocytes is transferrin [205,212]. Additionally, inflammation has been established rapidly to increase the synthesis of the iron regulatory hormone hepcidin [213], which inhibits the iron receptor ferroportin and thus controls the exclusion and tissue distribution of iron [10,204]. Moreover, other proteins involved in iron transportation or metabolism have been identified as being upregulated in and around WM lesions (e.g., divalent cation transporter 1, scavenger receptor class A, solute carrier family 11) [205,210]. In particular, transferrin and ferroportin binding are impaired, with more toxic hemosiderin and ferritin deposits having been detected, in the brains of MS patients [18,206,214]. The most important source of iron-related signals in cerebral T_2_-weighted MRI is thought to come from the ferric oxyhydroxide particles formed only in ferritin and hemosiderin [215].

However, iron is essential for physiological neuronal metabolism, including brain oxygen transport, electron transfer, neurotransmitter synthesis, and myelin production and maturation; its excessive accumulation, especially in pathologically affected brain tissue, is however harmful [17,208]. Poorly liganded iron generates free radicals via the Fenton reaction and thus catalyzes the increased production of reactive oxygen species leading to the oxidative damage of demyelinated axons [10,167,208]. This destruction is exacerbated by activated macrophages and microglia that release an excessive amount of pro-inflammatory mediators and cytokines (e.g., matrix metalloproteinases-9, tumor necrosis factor-α, interleukin-1β, interleukin-6) that can affect excitatory and inhibitory neurotransmission [17,120,210]. Thus, chronic inflammation can be invoked accompanied by oligodendrocytes apoptosis, lipid peroxidation, endothelial and blood–brain barrier damage, and further immune cell activation [10]. These features are present in MS, suggesting ferroptotic cell death, so-called ferroptosis, which is characterized by the presence of smaller mitochondria with ruptured membranes, reduced cristae, and the accumulation of lipid peroxidation products [19]. The hypothesis concerning the impact of iron deposits on MS progression is supported by an autopsy study in which an elevated iron accumulation in deep GM has been determined among various forms of MS (acute MS, RRMS, SPMS, and PPMS) [209]. Iron deposits occur in and around GM lesions [216,217] and in subcortical and deep GM of RRMS, SPMS, and PPMS patients [17,18,210]. However, iron accumulation has also been observed in deep GM (e.g., caudate nucleus, globus pallidus, and putamen) of early CIS patients [217,218], and increasing iron levels have been reported in the advancing stages of MS (CIS vs. RRMS [18], SPMS vs. RRMS [206]). Some studies have established that, although the basal ganglia have the highest iron content in healthy CNS [9,208], these iron deposits are significantly enlarged in MS [18,219]. If a higher iron accumulation is a sign of diffuse neurodegeneration, which is the cause of motor and cognitive dysfunction [18,206,209], then the consequent shortened T_2_ relaxation times of CNS structures can be considered as a biomarker of MS progression. Moreover, T_2_ shortening throughout the T_2_-hypointensities in GM has been shown to be more closely associated with neurologic status and cognitive impairment [217,220] than conventional MRI lesion assessment by means of lesion amount and volume [28,206]. This suggests that the evaluation of the T_2_-hypointensities in GM is the best predictor of whole-brain atrophy [18,28]. These data taken together indicate that iron chelation therapy could be applied in MS management [17,209,210]; however, many findings warn of its non-effectiveness [10]. Paradoxically, the cerebrospinal fluid and serum levels of iron are not elevated at all in MS patients [10,205,221]. As iron is indispensable for myelin synthesis (cofactor for enzymes enrolled in lipid biosynthesis), its deficiency may worsen the myelin regeneration that is so important for MS remission [204,221]. Furthermore, iron might paradoxically prevent oxidative damage via antioxidants such as cytochrome P450 or catalase (which contain iron-dependent heme groups) and nicotinamide adenine dinucleotide phosphate (NADPH; its synthesis requires iron-dependent ATP production) [10,222]. Therefore, iron supplementation or chelation should be critically evaluated based not only during MRI examination, but also during genetic and biochemical determinations.

### 4.3. Limitations of Parametric MR-Relaxation Mapping

Even though several assessment MR parameters (T_2_, T_2_*, T2’, T_2_ rho, R_2_ = 1/T_2_, R_2_* = 1/T_2_*, R_2_’= R_2_* − R_2_ [215]) have been used to improve the specificity and sensitivity of iron detection, only a weak correlation has been observed for WM iron accumulation [18,223,224]. This is most likely because of the influence of WM tissue water content (8–13% [193]), which moreover increases during various pathologic processes (i.e., inflammation, gliosis, edema, axonal demyelination) [17]. Furthermore, the decay of the MR visible proton signal in multi-echo T_2_ relaxation measurements is in general multiexponential, consisting of a very long relaxation associated with the free water in cerebrospinal fluid (T_2_ relaxation time >2000 ms), intermediate relaxation attributable to intra- and extracellular water (T_2_ relaxation time ~80 ms), and short relaxation typical for the water trapped between the myelin bilayers (T_2_ relaxation time ~30 ms) [27,225]. For in vivo tissue examination, a monoexponential fit of the signal decay is preferably used, resulting in a much easier evaluation of alterations in the brain tissue [193,199]. Moreover, several studies have reported limitations attributed to local changes in magnetic susceptibilities, magnetic field variation, and inhomogeneities that reduce the specificity for iron acquisition [18,208,226]. Unfortunately, despite the continuing development of MR relaxometry techniques (rotating frame and application of adiabatic pulses [227], gradient echo sampling of free induction decay and echo [228], asymmetric spin echoes [226], phase imaging [208], susceptibility-weighted imaging [18,205], magnetic field correlation imaging, and quantitative susceptibility mapping [215]), no consensus of one universal relaxometry method exists that enables the reliable quantification of iron in the brain without limitations [17]. In principle, this is because MR techniques do not detect iron directly, but only its manifestations, which might vary depending upon the surroundings and the magnetic state of the iron. This results in an increase in uncertainties and artifacts [17,208]. Theoretically, these difficulties can be suppressed by using complementary MR protocols [215,226,227,228], which are, however, time consuming, and require advanced data processing, which prevents their use in clinical practice.

## Figures and Tables

**Table 1:** Summary of general magnetic resonance imaging (MRI) diagnostic tools in multiple sclerosis (MS) assessment. Table summarizing general MRI diagnostic tools suitable for the assessment of MS with typical disease manifestations (marked with the symbol ~).

MRI	MS Assessment
**T_1_-weighted**	brain and spinal cord atrophy hypointensities (dark areas) ~ definitive distortion of the axonal structure, demyelination, neuronal loss, and edema
**Gd-enhanced T_1_-weighted**	hyperintensities (bright areas) ~ active inflammation; blood–brain barrier breakdown; active demyelination
**T_2_-weighted**	hyperintensities (bright areas) ~ edema, gliosis, demyelination; disease burden or lesion load
**FLAIR**	hyperintensities ~ demyelinated lesions; MS activity by reducing interference from the spinal fluid
**Magnetization transfer**	magnetization transfer ratio ~ degree of demyelination
**Diffusion**	hyperintensities, high diffusibility, low fractional anisotropy ~ demyelinated axons, and damaged nerve tracks
**Susceptibility-weighted**	hyperintensities ~ iron deposits
**Perfusion**	hyperintensities ~ acute inflammatory lesions hypointensities ~ tissue degradation
**Functional**	manifestation of disrupted brain activity

**Table 2:** Summary of MR techniques in MS assessment. Table summarizing MRS and other MR techniques suitable for MS assessment and typical disease manifestation.

MS Manifestation	MRS Techniques		Other MR Techniques
**demyelination**	**^1^H MRS:** ↑tCho **^31^P MRS:** ↑PDE, ↓[Mg^2+^]	↑tCho in DGM PDE > PME (mainly in WM) ↓[Mg^2+^] (in MS lesions)	MRI-T_2_ lesion load MR-relaxometry: ↑T_2_ magnetization transfer
**remyelination**	**^1^H MRS:** ↑tCho **^31^P MRS:** ↑PME	PME > PDE (mainly in WM)	
**neuro-axonal loss**	**^1^H MRS:** ↓tNAA, ↑Glx, ↓GABA **^31^P MRS:** ↑PCr, ↓ATP, ↓[Mg^2+^]	↓tNAA (in MS lesions, NAWM, cortical GM)↑Glx excitotoxicity (mainly in WM) ↓GABA (mainly in the hippocampus and sensorimotor cortex) ↑PCr and ↑PCr/β-ATP ↓[Mg^2+^] (mainly in WM)	diffusion: ↑ACD, ↓FA MRI: T_1_ hypointensities MRI: T_2_ hyperintensities MR-relaxometry: ↑T_2_
**gliosis, inflammation**	**^1^H MRS:** ↑tCho and ↑mIns	↑tCho (in MS lesions) ↑mIns (in MS lesions, NAWM)	perfusion: hyperintensities Gd-T_1_ hyperintensities
**cell debris accumulation**	**^1^H MRS:** ↑tCho **^31^P MRS:** ↑PDE	↑tCho (in MS lesions)	magnetization transfer
**cell energy failure**	**^31^P MRS:** ↑PCr, ↓ATP	↑PCr and ↑PCr/β-ATP (in acute MS lesions)	functional MRI
**BBB permeability**	unknown	unknown	Gd-T_1_ hyperintensities
**iron deposits**	unknown	unknown	MR-relaxometry: ↓T_2_ SWI: hyperintensities

Abbreviations: ACD: Apparent diffusion coefficient, ATP/mainly β-ATP: adenosine triphosphates, DGM: deep gray matter, FA: fractional anisotropy, GABA: γ-aminobutyric acid, Glx: glutamate and glutamine, [Mg^2+^]: intracellular Mg^2+^ concentration, mIns: myoInositol, NAWM: normal-appearing WM, PCr: phosphocreatine, PDE: phosphodiesters, PME: phosphomonoesters, SWI: susceptibility weighted imaging, tCho: total choline, tNAA: total N-acetyl-aspartate, WM: white matter; ↓: decreased, ↑: increased.
